# Fear of COVID-19, compliance with recommendations against virus transmission, and attitudes towards vaccination in Sweden

**DOI:** 10.1016/j.heliyon.2021.e08699

**Published:** 2021-12-29

**Authors:** A. Håkansson, E. Claesdotter

**Affiliations:** a Lund University, Faculty of Medicine, Department of Clinical Sciences Lund, Psychiatry, Lund, Sweden; b Region Skåne, Malmö Addiction Center, Clinical Gambling Disorder Unit, Malmö, Sweden; c Lund University, Faculty of Medicine, Department of Clinical Sciences Lund, Child and Adolescent Psychiatry, Lund, Sweden; d Region Skåne, Dept of Child and Adolescent Psychiatry, Lund, Sweden

**Keywords:** COVID-19, Pandemic, Vaccine, Recommendation, Fear of COVID scale, Mental health

## Abstract

Following the immense impact of the COVID-19 pandemic on health and everyday lives world-wide, people's fear of COVID-19 has been studied in a number of settings using the Fear of COVID scale. In Sweden, virus-preventing strategies have differed from comparable countries, with low use of formal lock-down procedures. It is crucial to study correlates of non-compliance with COVID-19 recommendations, and unwillingness to become vaccinated. This study aims to study whether fear of COVID is associated with mental distress and attitudes towards the pandemic, and to study correlates of non-compliance with key anti-COVID recommendations and with reluctancy to vaccination. This anonymous online survey study in web panel participants (N = 1,501) aimed to study a range of behavioral changes during COVID-19. Recommendations and vaccinations reluctancy were analyzed in logistic regressions against socio-demographic data, COVID-19 status, and mental health history. Internal consistency of the Fear of COVID scale was calculated. The Fear of COVID scale had a satisfactory internal consistency (Cronbach-alpha 0.84), and was significantly associated with compliance with all COVID-19 recommendations and with mental health. Non-compliance with recommendations was associated with low fear of disease and younger age, among other variables. Being against vaccination was associated, among other variables, with low fear of disease and with low education. In conclusion, the Fear of COVID scale appears to be associated with key attitudes towards the COVID-19 disease. Anti-virus strategies may need to promote compliance with recommendations in subgroups who feel low fear of disease or who believe not to be in a risk group for severe disease.

## 1 Introduction

The COVID-19 pandemic, caused by the global spread of the SARS-CoV-2 virus, has caused a vast disease burden, mortality and health consequences, including physical and mental disease, as well as extensive social problems (Holmes et al., 2020; Mansfield et al., 2021).

Like in many other countries in Europe and world-wide, the COVID-19 pandemic reached Sweden in the first months of 2020, with a first confirmed Swedish case reported on January 31, 2020. After a substantial first wave of COVID-19 transmission during the spring months of 2020, the summer months in Sweden were characterized by a substantial decrease in virus transmission, whereas a second wave started during the autumn of 2020. Here, data describing the general level of activity outdoors in Stockholm followed this pattern, and thereby indicated a compliance following the stricter recommendations introduced during the second wave (Rumpler et al., 2021). After only a partial decrease in virus transmission during the winter of 2020–2021, the first months of 2021 saw a third wave of virus transmission and again increased hospital-requiring disease burden in Sweden (Swedish Public Health Agency, 2021, https://fohm.maps.arcgis.com/apps/opsdashboard/index.html#/68d4537bf2714e63b646c37f152f1392).

Internationally, strategies from governments and authorities to decrease the spread of COVID-19 have included regulations or recommendation such as stay-at-home order, hand washing, travel bans, social distancing, and the wearing of face masks (Georgieva et al., 2021). Social distancing has been seen to decrease viral transmission and a number of measures of its consequences (Amiri, 2021). Factors behind the compliance with recommendations may be complex. Individual protective behavior has been shown to be predicted by fear of the disease (Jörgensen et al., 2021; Harper et al., 2020), lower susceptibility to disinformation about the pandemic (Roozenbeek et al., 2020), or by the belief that such interventions are effective (Clark et al., 2020). With respect to demographic correlates, men (Tomczyk et al., 2020; Clark et al., 2020) and younger individuals are less likely to comply with public anti-COVID-19 recommendations (Tomczyk et al., 2020; Wang et al., 2021a, Wang et al., 2021b; Berg-Beckhoff et al., 2021). In addition, it has been suggested that compliance with virus-preventive measures may also be affected by political beliefs (Grossman et al., 2020; Wang et al., 2021a, Wang et al., 2021b) and moral values (Díaz and Cova, 2021).

Reluctancy towards vaccination is a crucial challenge in the times of the COVID-19 pandemic, and data are emerging on its prevalence and correlates with respect to this specific infection. Also, there have been concerning data reporting that resistance against a potential vaccine appeared to increase with time during the first phases of the pandemic (Hyland et al., 2021). Willingness to become vaccinating has been examined in a number of studies, although most have rather assessed willingness to accept a hypothetical vaccine, before vaccines for COVID-19 were available (Reiter et al., 2020; Fisher et al., 2020: Khubchandani et al., 2021; Dodd et al., 2021). Early findings have indicated that being younger, and having less trust in science (Roozenbeek et al., 2020), female gender (Wang et al., 2021a, Wang et al., 2021b), lower level of education (Fisher et al., 2020: Khubchandani et al., 2021) or a perceived lower risk of getting infected (Khubchandani et al., 2021) are factors associated with vaccines reluctancy. A review paper prior to the availability of COVID-19 vaccines, demonstrated, among other factors, that a resistance to vaccines in general was associated with reluctancy also towards the COVID-19 vaccine, but more COVID-19-specific reasons were also involved, such as a hesitancy towards a vaccine being produced in rush during the pandemic, or a lower estimation of the severity of COVID-19 (Troiano and Nardi, 2021). Likewise, political identification and type of media consumption, and attitudes towards conspiracy theories, have been suggested to affect COVID-19 vaccine willingness (Ruiz and Bell, 2021).

Sweden has applied virus-protective strategies which have partly differed from those in most comparable countries (Mens et al., 2021; Yarmol-Matusiak et al., 2021). Specifically, no imperative lock-down measures or formal stay-at-home orders have been included in the national Swedish COVID-19 strategies, leaving, for example, elementary schools, restaurants, shopping malls and stores open throughout the pandemic.

A number of publications hitherto have assessed the fear of COVID-19 in the general population, using different psychometric instruments (Fitzpatrick et al., 2020). The Fear of COVID scale is a seven-item unidimensional scale that was developed early during the pandemic, and has been validated and translated into a number of languages (Ahorsu et al., 2020; Muller et al., 2021). The Fear of COVID scale has demonstrated a high level of internal consistency, and has been successfully validated against other mental health constructs such as anxiety and phobia. Hitherto, to the best of our knowledge, the scale has not been used in a Swedish setting (Muller et al., 2021).

Given the complexity in factors affecting compliance with COVID-19 regulations, it is of interest to study whether fear of the disease, as well as other variables, predict a high level of compliance with recommendations. The present study aimed to 1) to assess fear of COVID-19 in the Swedish population, and its potential association with psychological distress, belonging to a risk group, and with level of compliance with virus-preventing recommendations, and to 2) study predictors in the Swedish population of poor virus-preventing recommendation compliance, and with hesitation against COVID-19 vaccination. We hypothesized that fear of COVID-19 would be associated with a higher tendency to comply with anti-virus prevention strategies, and that it would be associated with a higher degree of mental distress. Also, we hypothesized that vaccine resistance would be more common in people with a low degree of fear of COVID-19, as well as with risk factors discussed in the literature, such as female gender, younger age, and lower education.

## 2 Method

The present study is a cross-sectional self-report online survey study, carried out in Sweden in the month of March, 2021, during the ongoing COVID-19 spread in the country. Respondents from the general population, aged 16 years and above, were invited from the online web survey panel of the market survey company Ipsos. Members of this web panel have enrolled voluntarily in order to receive market surveys, political opinion polls and similar surveys. The Ipsos web panel previously has been addressed in online surveys for research purposes related to problem gambling (Håkansson, 2020; Håkansson and Widinghoff, 2021). Respondents who complete a survey obtain merits which can thereafter be translated into goods or services bought, and with merits corresponding to around 1 Euro for a survey of the present extent. The survey is sent out to different age groups of the web panel, until a gender and age distribution close to that of the general population is achieved. The sampling method used in the previous paper, as in previous studies using the same company (Håkansson and Widinghoff, 2020a, 2020b), does not allow for a formal attrition analysis based on an originally intended sample, but instead gathers data until the intended number of completed answers (while respecting the intended gender and age distribution) is reached. In the present study, invitations were sent until a total of around 1,500 complete answers were obtained. The sample size aimed for was based on previous studies using the same type of data collection from the same market survey company, where representative samples of a smaller sample size have been able to address clinically relevant and statistically significant findings (Håkansson and Widinghoff, 2020a, 2020b). In addition, in the present study, the final distribution of age groups, gender, and geographical location (regions) was compared by Ipsos to the general population, such that the dataset was weighted according to a summarized weighting score derived from these three variables. When the survey was halted, the final sample consisted of 1,501 individuals.

The study was carried out from March 19 through March 29, 2021. Participants of the Ipsos web panel were invited with the information that the survey would address ‘computer gaming, gambling for money and other behavioral patterns in Sweden during COVID-19 – association with mental health, social situation and attitudes to the pandemic’. Thus, from the larger dataset collected, data focusing on behavioral addictions (gambling and gaming) during the pandemic are reported elsewhere. The survey was opened only in case the respondent provided electronic informed consent. The study was reviewed and approved by the Swedish Ethical Review Board (file number 2021/00369).

### 2.1 Variables

For the purpose of the present study, the Fear of COVID Scale (Ahorsu et al., 2020) was translated into Swedish. The translation was carried out by the first author, who translated directly from English, while also using the available Spanish (Martínez-Lorca et al., 2020), French (Mailliez et al., 2020), and Italian (Soraci et al., 2020) translations as points of reference. The scale consists of seven items describing different aspects of perceived fear of the disease, with responses ranges from ‘completely disagree’ (scored 1) to ‘completely agree’ (scored 5), thus with a final score ranging from 7 to 35. In the present study, the comparison to the scores describing psychological distress, and other factors such as the adaptation to different anti-COVID recommendations, included the summarized scale scores. When asking the questions of the scale in the present study, the respondents were instructed to think about the period in which the COVID-19 pandemic was the most intense in their region, based on the fact that virus transmission has differed substantially across geographical settings in Sweden during different phases of the pandemic. For the Fear of COVID scale, all questions were mandatory and therefore contained no missing data.

Psychological distress was measured using the Kessler-6 scale (Kessler et al., 2005), a six-item scale describing symptoms of depressed mood and symptoms of anxiety. This scale refers to symptoms perceived during the past six months. Here, questions could be skipped, leaving missing answers in 5–12 individuals for each separate item. Full scores were not available for 25 individuals who had wished not to answer at least one of the included items. In the analysis of dichotomized psychological distress (a total score of five or more), only 16 individuals had missing scores, after the scoring of respondents who fulfilled criteria of psychological distress (a total score of five or more) already from the available items.

Socio-demographic variables included were gender, age, level of education, and living conditions (which were dichotomized into living alone without children vs all options of living with partner and/or children or parents). These items were mandatory and therefore did not contain any missing data. Also, questions were asked about whether respondents had had a confirmed COVID-19 infection (‘yes’, ‘no’, or ‘do not know/prefer not to answer’), and about willingness to become vaccinated against COVID-19 (‘have been vaccinated’, ‘haven't been but wish to become vaccinated when I can’, ‘don't want to become vaccinated’, and ‘do not know/prefer not to answer’). Also, respondents were asked about whether they perceive themselves to belong to a risk group of becoming severely ill in COVID-19 (‘yes’, ‘no’, or ‘do not know/prefer not to answer’). Here, all questions were mandatory and therefore had no missing answers, whereas the option ‘do not know/prefer not to answer’ was dealt with as a missing answer in the analyses.

The following variables described COVID-19 compliance with recommendations; recommendation not to meet new people, not to gather in stores and shopping malls and similar, not to travel to other regions or countries, and to stay at home in case of minor symptoms. These questions were answered with the options ‘to a very high degree’, ‘to a fairly high degree’, ‘to a fairly low degree’, and ‘to a very low degree’, along with the option ‘do not know/prefer not to answer’. A general question was asked about whether the individual had followed recommendations ‘more than others’, ‘less than others’, ‘approximately like others’, as well as ‘don't know/prefer not to answer’. These recommendations were chosen as they are likely to have been well-known to the general public during a substantial proportion of the COVID-19 pandemic. As face masks has not been mandatory in Sweden but only a recommendation in specific circumstances, this was not included in the study. Questions about recommendations were mandatory, with no missing answers, but the responses ‘do not know/prefer not to answer were not included in the statistical analyses.

### 2.2 Setting

With respect to virus-preventing strategies, several researchers have reported that Sweden has differed from a large number of other settings, most importantly due to the absence of full stay-at-home orders or lockdown closures in Sweden (Baral et al., 2020; Farina and Lavazza, 2020; Yarmol-Matusiak et al., 2021). A large difference in fatal COVID-19 cases has been seen between Sweden and neighbor Nordic countries with lower death rates (Rypdal et al., 2021).

The first confirmed case of COVID-19 in Sweden was reported on January 31, 2020, and the first fatality on March 11, 2020. Government announcements of COVID-19-preventing strategies were made in mid-March 2020, and after a peak number of deaths during the first part of April, transmission and mortality decreased, with low values during the summer months and a bottom level of deaths in late August, 2020. After a gradual increase of cases in the early autumn, a clear ‘second wave’ increase led to more extensive recommendation in an increasing number of regions, starting in mid-October, and the second wave, measured as virus cases or deaths, peaked in December, 2020. After only a partial decrease of cases, a third wave started with a new increase in virus transmissions and hospitalizations from the month of February, where mortality rates remained stable after the partial decrease in the start of 2021. Altogether, the peak number of intensive care-treated COVID-19 patients were reported, for the first, second and third waves, respectively, on April 22, 2020, December 29, 2020, and April 8, 2021 (Swedish Public Health Agency, 2021, https://fohm.maps.arcgis.com/apps/opsdashboard/index.html#/68d4537bf2714e63b646c37f152f1392). The first dose of COVID-19 vaccine in Sweden was administered on December 27, 2020. Thus, the present study was carried out during the third wave of severe viral transmission in Sweden, and when around 6–8 percent of the adult population had been fully vaccinated, and when around 16–18 percent had received at least one dose of vaccine (Swedish Public Health Agency, 2021, https://fohm.maps.arcgis.com/apps/opsdashboard/index.html#/3e20f6433c14468f8fecb177ab315b9d).

### 2.3 Statistical methods

The reporting of prevalence measures and group-wise comparisons relate to the weighted data, whereas logistic regression analyses were carried out using the unweighted data. Logistic regressions were carried out with each of the recommendations as the dependent variable, in order to study variables associated with a non-adherence with the recommendation (admitting adhering to the recommendation to a low or very low degree). Also, a separate logistic regression analysis was run for unwillingness to become vaccinated. In all logistic regression analyses, individuals with missing data on any of the items were excluded. In these analyses, the following variables were used as independent variables potentially associated with non-adherence or unwillingness to become vaccinated fear of COVID: gender, age in increasing age groups, level of education (post-high-school education vs lower), living conditions (alone without children vs all others), occupational status (working/studying vs all others), and risk group status (yes vs others). In the analysis of vaccine reluctancy, variables were also controlled for whether respondents had had a COVID-19 infection or not (yes vs others).

For the Fear of COVID scale, the Swedish version, Cronbach-alpha was calculated for internal consistency. Fear of COVID-19 was analyzed for potential associations with the survey items describing self-perceived status as a risk group in case of COVID-19 infection, and with the different measures of compliance with virus-preventing recommendations. In addition, a correlation with past-six-month psychological distress was reported as correlations with the full Kessler-6 score, as well as with the individual score of each Kessler-6 item.

For all statistical analyses, SPSS version 25.0 was used (IBM, SPSS).

## 3 Results

### 3.1 Sample description, COVID-19 status, vaccination and risk group status

The descriptive data of the sample are shown in Table 1 (weighted data). Fifty-one percent of the respondents were men, and 49 percent were women.

**Table 1:** Sample characteristics. Weighted data. N = 1,501.

	% (n)
Gender	
- Female	49 (740)
- Male	51 (761)
Age (years)	
- 16-17	2 (35)
- 18-24	11 (162)
- 25-29	11 (163)
- 30-39	16 (237)
- 40-49	12 (182)
- 50-59	21 (311)
- 60-69	11 (160)
- 70 or older	17 (251)
Living conditions	
- Living with partner and children	24 (353)
- Living with partner without children	38 (566)
- Living alone with children	5 (74)
- Living alone without children	26 (387)
- Living with my parents	8 (121)
Occupation	
- Studying	13 (198)
- Working	54 (815)
- Job-seeking	5 (74)
- Short-term unemployed due to COVID-19	1 (10)
- Retired	24 (364)
- Other	3 (40)
Level of education	
- Elementary school	9 (130)
- High school	39 (585)
- Post high school studies without full exam	17 (249)
- Post high school studies, full exam	33 (489)
- Other	3 (49)

Eleven percent of respondents (n = 167) reported having had a confirmed COVID-19 infection, whereas 86 percent (n = 1,294) had not, and three percent (n = 40) did not now or preferred not to answer. Ten percent (n = 155) had been vaccinated, 73 percent (n = 1,091) wished to become vaccinated, and eleven percent (n = 163) reported that they did not want to become vaccinated. Six percent (n = 91) did not know or preferred not to answer. A total of 24 percent (n = 364) of respondents perceived themselves to belong to a risk group with respect to COVID-19, whereas 68 percent (n = 1,022) did not, and eight percent (n = 115) did not know or preferred not to answer.

### 3.2 Compliance with recommendations (weighted data)

Thirty-eight percent (n = 570) reported that they had followed recommendations more than most others, 56 percent (n = 842) like others, five percent (n = 73) less than most others, and one percent (n = 16) did not know or preferred not to answer.

The recommendation not to be around new people had been followed by 44 percent (n = 666) to a very high degree, by 44 percent (n = 656) to a fairly high degree, eight percent (n = 117) to a fairly low degree, three percent (n = 46) to a very low degree, and one percent (n = 15) did not know or preferred not to answer. The recommendation to avoid gathering in stores and similar had been followed by 44 percent (n = 665) to a very high degree, by 45 percent (n = 670) to a fairly high degree, eight percent (n = 122) to a fairly low degree, two percent (n = 33) to a very low degree, and one percent (n = 11) did not know or preferred not to answer.

The recommendation to remain at home in case of any minor symptom had been followed by 66 percent (n = 992) to a very high degree, by 25 percent (n = 370) to a fairly high degree, five percent (n = 71) to a fairly low degree, two percent (n = 33) to a very low degree, and two percent (n = 35) did not know or preferred not to answer. The recommendation to avoid travelling to other regions or countries had been followed by 66 percent (n = 985) to a very high degree, by 25 percent (n = 378) to a fairly high degree, six percent (n = 90) to a fairly low degree, two percent (n = 34) to a very low degree, and one percent (n = 14) did not know or preferred not to answer.

### 3.3 Fear of COVID scale (weighted data)

The Cronbach-alpha value for the Fear of COVID scale was 0.84. The overall fear of COVID score (weighted data) was, on average, 14.47 (SD 5.64, median 14, inter-quartile range 10–18, 90^th^ percentile 22). Fear of COVID scale scores were higher in women (M = 15.89, SD = 5.93) than in men (M = 13.10, SD = 4.98), t(1,441.5) = 9.86, p < .001), and higher in individuals who reached the cut-off for psychological distress (M = 15.77, SD = 5.92) than in respondents who scored below cut-off for psychological distress (M = 12.91, SD = 4.78), t(1,468.6) = -10.30, p < .001. A correlation analysis between the total score of the Fear of COVID score, and the total Kessler-6 score for psychological distress, demonstrated a weak correlation (r = 0.28), analyzed for the 1,477 respondents with full Kessler-6 data. The correlation between the Fear of COVID score and each separate Kessler-6 item was the highest for nervousness (r = 0.29) and hopelessness (n = 0.29), lower for depressive symptoms (r = 0.23), for the feeling that everything is an effort (n = 0.20) and for worthlessness (p = 0.20), and the lowest for restlessness (p = 0.16).

Fear of COVID was significantly higher in individuals who were not working/studying (p < 0.001), who had not had a COVID-19 infection (p < 0.01), who had ever been prescribed mental health treatment (p < 0.001), who had been or wanted to become vaccinated (p < 0.001), who believed to belong to a risk group (p < 0.001), who believed they followed recommendation like others or more (p < 0.001), and who followed each of the recommendations included in the study (p < 0.001 for all, except for p = 0.02 for the recommendation to remain at home in case of symptoms). However, the fear of COVID score was not associated with level of education (p = 0.51), or living conditions (p = 0.90, Table 2). Also, the fear of COVID score did not differ between those who already had been vaccinated and those who wanted to become vaccinated (p = 0.33).

**Table 2:** Fear of COVID scale scores – associations with demographic data, COVID-19 status, and vaccination willingness and compliance with recommendations.

	Fear of COVID-19 scale score, average	*p* value
Gender		
- Female (n = 740)	15.89	<0.001
- Male (n = 761)	13.10	
Education		
- Above high school (n = 737)	14.38	0.51
- High school or lower (n = 764)	14.57	
Occupation		
- Studying/working (n = 1,013)	14.09	<0.001
- Other (n = 488)	15.28	
Living conditions		
- Alone without children (n = 387)	14.51	0.90
- Other (n = 1,114)	14.46	
Ever prescribed mental health treatment		
- Yes (n = 357)	15.64	<0.001
- No (n = 1,144)	14.11	
Had COVID-19		
- Yes (n = 167)	13.39	<0.01
- No (n = 1,334)	14.61	
Perceived to be in risk group		
- Yes (n = 364)	16.63	<0.001
- No (n = 1,137)	13.78	
Attitudes to vaccination^1^		
- Against (n = 163)	12.68	<0.001
- Vaccinated or wants vaccine (n = 1,247)	14.57	
Vaccination status in those with favorable attitudes towards vaccination		
- Already vaccinated (n = 155)	14.99	0.33
- Wants to become vaccinated (n = 1,091)	14.51	
Follows restrictions^1^		
- Less than others (n = 73)	11.75	<0.001
- Like others or more (n = 1,412)	14.60	
Respects travel recommendations^2^		
- Yes (n = 1,363)	14.65	<0.001
- No (n = 124)	12.76	
Respects recommendation to stay at home in case of symptoms^2^		
- Yes (n = 1,362)	14.59	0.02
- No (n = 104)	13.16	
Respects recommendation not to meet people^2^		
- Yes (n = 1,323)	14.73	<0.001
- No (n = 163)	12.57	
Respects recommendation to avoid gatherings in stores etc^2^		
- Yes (n = 1,335)	14.78	<0.001
- No (n = 154)	11.66	

1 Excludes those who don't know or prefer not to answer.

2 ‘Yes’ includes individuals reporting to follow this recommendation to a ‘high’ or ‘fairly high’ degree, vs individuals following this recommendation to a ‘low’ or ‘fairly low’ degree (excluded those who don't know or prefer not to answer).

### 3.4 Multivariate analyses of compliance to recommendations and vaccination (unweighted data)

In logistic regression, based on unweighted data (n = 1,486), failure to comply with the recommendation not to meet new people was associated with lack post high school education (OR 0.58 [0.40–0.83], p < 0.01), age (OR 0.77 [0.69–0.85], p < 0.001), and low fear of COVID (OR 0.93 [0.89–0.96], p < 0.001), but not with gender (p = 0.45), living conditions (p = 0.82), occupational status (p = 0.79), and belonging to a risk group (p = 0.20).

In logistic regression (n = 1,490), failure to comply with the recommendation against gathering was significantly associated with lack of post high school education (OR 0.58 [0.40–0.84], p < 0.01), age (OR 0.76 [0.69–0.85], p < 0.001), and low fear of COVID (OR 0.89 [0.85–0.92], p < 0.001), but not with gender (p = 0.75), living conditions (p = 0.68), occupational status (p = 0.53), and belonging to a risk group (p = 0.75).

In logistic regression (n = 1,464), failure to comply with the recommendation to remain at home in case of symptoms was significantly associated with male gender (OR 2.28 [1.47–3.55], p < 0.001), and age (OR 0.87 [0.77–0.99], p = 0.04), whereas it was not associated with level of education (p = 0.46), living conditions (p = 0.63), occupational status (p = 0.74), belonging to a risk group (p = 0.44), and fear of COVID (p = 0.27).

In logistic regression (n = 1,487), failure to comply with the recommendation not to travel was significantly associated with male gender (OR 1.58 [1.05–2.36], p = 0.03), age (OR 0.82 [0.73–0.92], p < 0.01), and low fear of COVID (OR 0.95 [0.91–0.99], p = 0.01), but not with level of education (p = 0.49), living conditions (p = 0.71), occupational status (p = 0.68), and it was marginally associated with not belonging to a risk group (p = 0.05).

In logistic regression (n = 1,486), following recommendations less than others was significantly associated with age (OR 0.83 [0.72–0.97], p = 0.02), and low fear of COVID (OR 0.89 [0.84–0.95], p < 0.001), but not associated with gender (p = 0.41), level of education (p = 0.63), living conditions (p = 0.21), occupational status (p = 0.95), and risk group status (p = 0.88).

In logistic regression (n = 1,410), being against vaccination was significantly associated with lack of post high school education (OR 0.66 [0.46–0.94], p = 0.02), living alone (OR 1.56 [1.08–2.25], p = 0.02), not belonging to a risk group (OR 0.49 [0.28–0.86], p = 0.01), low fear of COVID (OR 0.93 [0.90–0.97], p < 0.001), younger age (OR 0.85 [0.76–0.94], p < 0.01), and female gender (OR 1.46 [1.02–2.07], p = 0.04), but not with occupational status (p = 0.44). When controlling for COVID-19 status, the same variables remained significant, with the addition of COVID-19 status, which was negatively associated with being against a vaccination (OR 0.40 [0.20–0.81], p = 0.01).

## 4 Discussion

The present study examined fear of COVID-19 in the Swedish population, as well as compliance with virus-preventing recommendations and willingness to become vaccinated. First, the fear of COVID scale in its Swedish version demonstrated high internal consistency, and was associated, among other factors, with mental distress and with compliance with a number of recommendations. Second, key correlates of compliance with COVID-19-related recommendations and vaccination willingness were identified. In adjusted analyses, non-compliance was overall associated with low fear of disease and with younger age. Men were more likely not to remain at home in case of minor symptoms, and more likely not to adhere to advice against travelling. Lower education was not associated with all non-compliance measures, but with failure to adhere to advice not to meet people and gather in stores and similar. Reluctance to become vaccinated was more common in the young, in those with low education or living alone, and associated with low fear of disease and with already having had the infection.

The present study is the first – to the best of the authors’ knowledge – to publish Swedish data describing fear of COVID-19. Internal consistency (Cronback-alpha) in the present study was close to that of the original publication of the scale (0.88, Ahorsu et al., 2020), and, for example, in the Israeli population (0.82, Bitan et al., 2020), in the Italian population (0.87, Soraci et al., 2020), and in Spanish students (0.86, Martínez-Lorca et al., 2020). In a recent review paper summarizing 15 papers assessing the Fear of COVID scale, a number of studies identified significant correlations between the scale and phobia or anxiety (Muller et al., 2021). Also, in the present study, an association was seen with a history of mental health problems requiring a prescription for treatment. However, the association with screening status for recent psychological distress, measured with the Kessler-6 items, was significant but relatively weak. It is possible that the relative weakness of this association is due to the fact that the Fear of COVID measure referred to the periods most affected by the pandemic, whereas the mental health screen referred specifically to the past six months. At least, from the present data in Sweden, it can be concluded that the Fear of COVID scale is most likely associated with a mental health disease history, and to some extent associated with how respondents in Sweden endorsed mental health symptoms during or between the second and third COVID-19 waves in the country. Altogether, the study lends support to the use of the Fear of COVID scale in Sweden.

In addition, the study provides evidence of possible explanations of poor adherence with virus-preventing strategies, and also suggests a number of factors less likely to affect such patterns of adherence. As expected, individuals who perceived lower fear of the infection were more likely not to adhere to recommendations. The only compliance variable assessed which was independent of fear was the failure to comply with the advice to remain at home in case of even minor symptoms of disease, an advice strongly and frequently communicated from Swedish authorities. While the non-compliance for this advice did not prove to be associated with fear of COVID, this may be due to the fact that this recommendation is perceived as aimed to decrease spread of the infection to others, rather than the opposite. Thus, intuitively, being afraid of catching the COVID-19 virus may not necessarily lead an individual to adhere to a recommendation rather focusing on not spreading the virus further (in contrast to a recommendation perceived to protect oneself against the contamination from others). The latter raises the issue of how to promote compliance with the recommendation in sub-groups of the population who feel a low degree of fear of the disease for themselves, possibly thanks to young age and good health conditions, and although the recommendations may rather be intended to prevent virus transmission to others. This is in line with previous data reporting that self-isolating during COVID-19 is markedly more often described as a means of protecting oneself from the disease, rather than in order to prevent the spreading to others (Leigh et al., 2020). Likewise, anti-COVID recommendations may aim to reduce both individuals’ risk of catching the disease, as well to reduce virus transmission overall and thereby the risk of others. It has been suggested that these two separate aims, to protect oneself and to reduce virus transmission overall, may be driven by diverse psychological processes (Liekefett and Becker, 2021).

The association of fear and compliance with anti-COVID recommendations may not seem surprising (Demitras-Madran, 2021). How people perceive the pandemic is likely to affect their degree of compliance with restrictions against it (Zajenkowski et al., 2020). Previous literature has demonstrated an association between fear of the disease and compliance (Segal et al., 2021), such that for example, adherence to lockdown restrictions may be associated with the level of perceived threat from the pandemic (Bonetto et al., 2021).

Results from a web survey carried out during the first wave of the pandemic, i.e. relatively early in the progress of the disease showed that personal use of protective strategies are associated with self-efficacy. In addition to fear of the disease, interestingly, it was also demonstrated that trust in others and trust in institutions had a limited role in individuals’ protective measures (Jörgensen et al., 2021). This may corroborate with the findings of the present study with respect to the recommendation about not to leave home in case of symptoms, even lighter symptoms. Thus, if individuals do not feel afraid of the infection themselves, trust in institutions and their recommendations may not be enough to adhere with these.

The present study did not include questions about wearing a face mask. Throughout the pandemic, the wearing of face masks has never been mandatory in Sweden. Authority recommendations about face masks were issues during the second wave, i.e. relatively late into the pandemic, and limited only to rush hours in public transports and other situations of public gatherings. For this reason, questions about mask wearing were not included, as most people in Sweden may not perceive themselves to be subject to a mask recommendation. Thus, attitudes towards mask wearing would have been harder to assess than in other settings, where the face mask wearing has been more generally applied and with stricter regulations. It has been shown that people wearing a face mask are perceived to prefer larger social distancing (Seres et al., 2021), theoretically, the signaling of a need to maintain distance may be rarer in setting where few individuals wear a face mask.

For several items assessing recommendation non-compliance, this was associated with younger age. This is consistent with previous research indicating that young age is associated with poor anti-COVID-19 compliance (Tomczyk et al., 2020). Male gender was associated with non-compliance to remain at home in case of symptoms, and with non-compliance with travel restrictions. Again, male gender has previously been associated with low COVID-19 measure compliance (Tomczyk et al., 2020). In an early study during COVID-19, social media-recruited adults from North America and Europe, male gender and younger age were risk factors of poor adherence to social distancing regulation (Coroiu et al., 2020). Other studies have also reported lower compliance with anti-virus recommendations in men and in the young (Solomou and Constantinidou, 2020), well in line with the present study.

Overall, compliance with regulation may also be associated with more detailed psychological factors, which are more difficult to address in a brief web survey. For example, Carvalho and co-workers (2020) demonstrated, in Brazilian adults, that low extroversion and high conscientiousness favor adherence to anti-COVID-19 measures. Likewise, although its quantitative impact may be uncertain, it also cannot be excluded that conspiracy theories, affecting the perception of severity of the pandemic situation in sub-groups of individuals, may have a negative effect on compliance (Roser and Jamieson, 2020; Freeman et al., 2020). Likewise, political beliefs have been shown to affect compliance with recommendations in some settings (Grossman et al., 2020).

In the present study, reluctance towards vaccination was, expectedly, associated with a low fear of COVID-19, and with the reporting of having had the infection already. Also, reluctance towards vaccination was more common in the young, and in individuals living alone and reporting lower level of education. Expectedly, it has been reported that hypothetical willingness to be vaccinated is associated with the belief of a higher risk of catching the disease (Reiter et al., 2020). The link between vaccine reluctancy and younger age, as well as the perception of not belonging to a risk group, is consistent with previous data, such as an online study in Japan showing higher willingness for vaccination in older individuals and those with more severe underlying conditions (Yoda and Katsuyama, 2021), the latter possibly similar to a notion of being in a risk group. Also, early 2020 data from Australia demonstrated that resistance against a possible COVID-19 vaccine (unavailable at the time) was associated with a belief that COVID-19 dangers were exaggerated, and with lower level of education (Dodd et al., 2021). The same association with lower education was seen in US samples examined with respect to a hypothetical COVID-19 vaccination (Fisher et al., 2020; Khubchandani et al., 2021). These findings are consistent with the present study, where low education was one of the factors associated with vaccine reluctancy. Likewise, a more recent US study highlighted socio-demographic factors, including lower education, as being associated with vaccine hesitancy (Killgore et al., 2021).

Reasons for not accepting the vaccine often appear to be related to safety issues, such as concerns about side effects, or due to a low fear of the COVID-19 disease (Neumann-Böhme et al., 2020). A particular feature of the present study is that it was carried out while vaccination was actively ongoing in the country; survey responses were provided around 2.5–3 months after the very first vaccine dose was delivered in Sweden. However, most respondents in the present study were likely not in the population groups prioritized for vaccination up to the dates when the survey was carried out, except in case they were employed in health care sectors. However, as the study was carried out when vaccines were already available, it thereby differs from a number of studies assessing potential future vaccines in the earlier phases of the pandemic. Fear of severe side effects, although rare, may potentially have been influenced by regulatory and policy discussions around side effects of specific vaccines during the first months of 2021. These have included, for example, the few cases of severe coagulopathies reported after COVID-19 vaccination (Parums, 2021). Few studies have been conducted while vaccinations have been actively available. In health care workers in Canada, acceptance of an offered vaccination was associated with being older, and with exposure to the infection in their work. As in other research, the fact that the vaccine is new and leaves limited time for consideration, are listed as factors associated with non-acceptance (Dzieciolowska et al., 2021).

The present study involves a number of potential limitations. First, the present data were based on a web panel survey, and although the study sample was intended and weighted to represent the general population, it is hard to know whether the choice to enroll in a web panel may be associated with other characteristics and, in this case, with other attitudes towards the pandemic than for their peers in the general population. Also, the study was part of an overall focus on behavioral change during the pandemic. In that sense, it should be borne in mind that web panel members may have become more interested in entering the survey because of another content of the e-mail invitation, such as the part involving computer gaming and gambling. Online screen behaviors, including these potential problem behaviors, are among those most commonly addressed in the media and research community during the pandemic, such that the addressing of those specific issues may not cause such a large bias. In addition, the format of a web survey does not allow for more in-depth questions and necessarily reports data in a briefer way and more summarized than, for example, if interviews would have been conducted face to face, or even in a qualitative study design. For example, as in previous studies on fear of COVID-19 in general population surveys (Muller et al., 2021), formal mental health diagnoses could not be established, again, due to the brief format and the lack of more in-depth interviews. On the other hand, the present study used the Kessler-6 scale as a well-established screen for mental distress, in addition to a brief item describing previous need for mental health treatment. Another limitation is that the time frame of the Kessler-6 measure may differ from the period in which the fear of COVID-19 was the most pronounced, as the latter could vary depending on regional virus transmission or people's varying risk of exposure in their everyday lives. Here, further studies may be needed, focusing more on the in-depth analyses of factors behind behavioral change and failure to adhere to authority recommendations during the pandemic. Also, the fear of COVID-19 questions referred to the period of most intense virus transmission (as this may differ regionally). Thus, results could not demonstrate – and could not be expected to demonstrate – a difference in fear of COVID-19 between those already vaccinated and those who intended to become vaccinated, and therefore, the study was not designed to demonstrate whether the fear of the disease would decrease when an individual was provided with the vaccine.

## 5 Conclusions

The present study, in an online web panel, supports the use of a Swedish translation of the Fear of COVID scale, and demonstrates an association between fear of COVID-19 and the individual perception of belonging to a risk group, previous mental health history, and adherence to government recommendations and willingness to vaccination. For a number of government recommendations, and when controlling for a number of potential correlates, individuals who reported non-compliance with the regulations were younger, reported less fear of disease, and had lower level of education. Men were more likely than women to report non-compliance with the recommendation to stay at home in case of symptoms, and with travel restrictions. Interventions may be needed in order to promote vaccination in individuals who have already had the COVID-19 disease or who feel less fear of the disease, as well as in the young and in individuals with lower education, where vaccination willingness was lower.

## Declarations

### Author contribution statement

Håkansson A: Conceived and designed the experiments; Performed the experiments; Analyzed and interpreted the data; Contributed reagents, materials, analysis tools or data; Wrote the paper.

Claesdotter E: Analyzed and interpreted the data; Wrote the paper.

### Funding statement

Anders Håkansson was supported by Region Skåne hospital organization and by Svenska Spel.

### Data availability statement

The authors do not have permission to share data.

### Declaration of interests statement

The authors declare the following conflict of interests: Both authors have received funding from the state-owned gambling operator of Sweden (AB Svenska Spel) and from its research council. Håkansson has received funding from the research council of the alcohol monopoly of Sweden. None of these organizations were involved in the present work in any way.

### Additional information

No additional information is available for this paper.
